# Hybrid genome assembly and annotation of *Danionella translucida*

**DOI:** 10.1038/s41597-019-0161-z

**Published:** 2019-08-26

**Authors:** Mykola Kadobianskyi, Lisanne Schulze, Markus Schuelke, Benjamin Judkewitz

**Affiliations:** Einstein Center for Neurosciences, NeuroCure Cluster of Excellence, Charité – Universitätsmedizin Berlin, Charitéplatz 1, 10117 Berlin, Germany

**Keywords:** Neuroscience, Model vertebrates, Next-generation sequencing, Sequence annotation

## Abstract

Studying neuronal circuits at cellular resolution is very challenging in vertebrates due to the size and optical turbidity of their brains. *Danionella translucida*, a close relative of zebrafish, was recently introduced as a model organism for investigating neural network interactions in adult individuals. *Danionella* remains transparent throughout its life, has the smallest known vertebrate brain and possesses a rich repertoire of complex behaviours. Here we sequenced, assembled and annotated the *Danionella translucida* genome employing a hybrid Illumina/Nanopore read library as well as RNA-seq of embryonic, larval and adult mRNA. We achieved high assembly continuity using low-coverage long-read data and annotated a large fraction of the transcriptome. This dataset will pave the way for molecular research and targeted genetic manipulation of this novel model organism.

## Abstract (summary)

**Table:** 

Design Type(s)	sequence assembly objective • sequence annotation objective • transcription profiling design
Measurement Type(s)	transcription profiling assay • genome
Technology Type(s)	RNA sequencing • DNA sequencing
Factor Type(s)	age
Sample Characteristic(s)	Danionella translucida • whole body

Machine-accessible metadata file describing the reported data (ISA-Tab format)

## Background & Summary

**Fig. 1:**
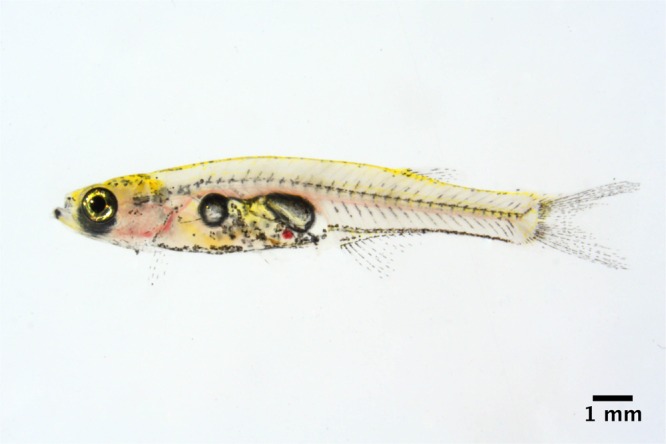
Male adult *Danionella translucida* showing transparency.

The size and opacity of vertebrate tissues limit optical access to the brain and hinder investigations of intact neuronal networks *in vivo*. As a result, many scientists focus on small, superficial brain areas, such as parts of the cerebral cortex in rodents, or on early developmental stages of small transparent organisms, like zebrafish larvae. In order to overcome these limitations, *Danionella translucida* (DT), a transparent cyprinid fish^1,2^ with the smallest known vertebrate brain, was recently developed as a novel model organism for the optical investigation of neuronal circuit activity in vertebrates^3,4^. The majority of DT tissues remain transparent throughout its life (Fig. 1). DT displays a variety of social behaviours, such as schooling and vocal communication, and is amenable to genetic manipulation using genetic tools that are already established in zebrafish. As such, this species is a promising model organism for studying the function of neuronal circuits across the entire brain. Yet, a continuous annotated genome reference is still needed to enable targeted genetic and transgenic studies and facilitate the adoption of DT as a model organism.

Next-generation short-read sequencing advances steadily decreased the price of the whole-genome sequencing and enabled a variety of genomic and metagenomic studies. However, short-read-only assemblies often struggle with repetitive and intergenic regions, resulting in fragmented assembly and poor access to regulatory and promoter sequences^5,6^. Long-read techniques, such as PacBio and Nanopore, can generate reads up to 2 Mb^7^, but they are prone to errors, including frequent indels, which can lead to artefacts in long-read-only assemblies^6^. Combining short- and long-read sequencing technologies in hybrid assemblies recently produced high-quality genomes in fish^8,9^.

Here we report the hybrid Illumina/Nanopore-based assembly of the *Danionella translucida* genome. A combination of deep-coverage Illumina sequencing with a single Nanopore sequencing run produced an assembly with scaffold N50 of 340 kb and Benchmarking Universal Single-Copy Orthologs (BUSCO) genome completeness score of 92%. Short- and long-read RNA sequencing data used together with other fish species annotated proteomes produced an annotation dataset with BUSCO transcriptome completeness score of 86%.

## Methods

### Genomic sequencing libraries

For genomic DNA sequencing we generated paired-end and mate-pair Illumina sequencing libraries and one Nanopore library. We extracted DNA from fresh DT tissues with phenol-chloroform-isoamyl alcohol. For Illumina sequencing, we used 5 days post fertilisation (dpf) old larvae. A shotgun paired-end library with 500 bp insert size was prepared with TruSeq DNA PCR-Free kit (Illumina). Sequencing on HiSeq 4000 generated 1.347 billion paired-end reads. A long ~10 kb mate-pair library was prepared using the Nextera Mate Pair Sample Prep Kit and sequenced on HiSeq 4000, resulting in 554 million paired-end reads. Raw read library quality was assessed using FastQC v0.11.8^10^.

A Nanopore sequencing high-molecular-weight gDNA library was prepared from 3 months post fertilisation (mpf) DT tails. We used 400 ng of DNA with the 1D Rapid Sequencing Kit (SQK-RAD004) according to manufacturer’s instructions to produce the longest possible reads. This library was sequenced with the MinION sequencer on a single R9.4 flowcell using MinKNOW v1.11.5 software for sequencing and base-calling, producing a total of 4.3 Gb sequence over 825k reads. The read library N50 was 11.6 kb with the longest read being approximately 200 kb. Sequencing data statistics are summarised in Table 1.

**Table 1:** Sequencing library statistics.

*Illumina paired*-*end gDNA*	
Number of reads	1.347 × 10^9^
Total library size	136.047 Gb
Insert size	500 bp
Read length	2 × 101 bp
Estimated coverage	186×
*Illumina mate*-*pair gDNA*	
Number of reads	554.134 × 10^6^
Total library size	55.968 Gb
Insert size	10 kb
Read length	2 × 101 bp
Estimated coverage	77×
*Nanopore gDNA*	
Number of reads	824.880 × 10^3^
Total library size	4.288 Gb
Read length N50	11.653 kb
Estimated coverage	5.8×
*Nanopore cDNA*	
Number of reads	208.822 × 10^3^
Total library size	279.584 Mb
Read length N50	1.812 kb
*BGI 3 dpf larvae mRNA*	
Number of reads	130.768 × 10^6^
Total library size	13.077 Gb
Read length	2 × 100 bp
*BGI adult mRNA*	
Number of reads	128.546 × 10^6^
Total library size	12.855 Gb
Read length	2 × 100 bp

gDNA stands for genomic DNA sequencing, cDNA for reverse-transcribed complementary DNA, mRNA for poly-A tailed RNA sequencing.

### Genome assembly

**Fig. 2:**
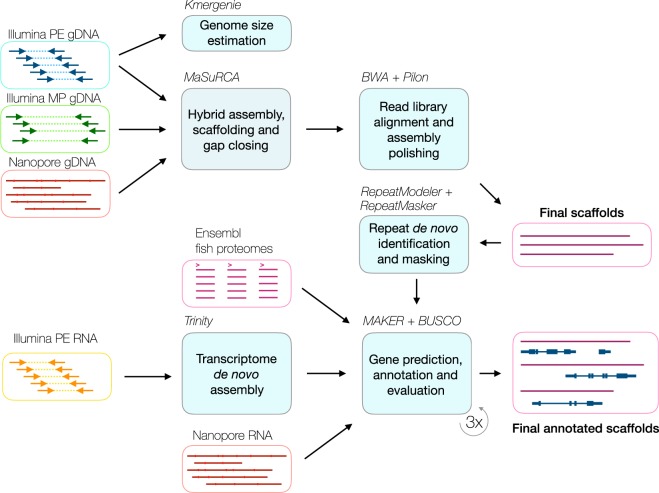
DT genome assembly and annotation pipeline. **PE**, paired-end; **MP**, mate-pair.

The genome assembly and annotation pipeline is shown in Fig. 2. We estimated the genome size using the k-mer histogram method with Kmergenie v1.7016 on the paired-end Illumina library preprocessed with fast-mcf v1.04.807^11,12^, which produced a putative assembly size of approximately 744 Mb. This translates into 186-fold Illumina and 5.8-fold Nanopore sequencing depths.

Multiple published assembly pipelines utilise a combination of short- and long-read sequencing. Our assembler of choice was MaSuRCA v3.2.6^13^, since it has already been used to generate high-quality assemblies of fish genomes^8,9^, providing a large continuity boost even with low amount of input long reads^14^. Briefly, Illumina paired-end shotgun reads were non-ambiguously extended into the superreads, which were mapped to Nanopore reads for error correction, resulting in megareads. These megareads were then fed to the modified CABOG assembler that assembles them into contigs and, ultimately, mate-pair reads were used to do scaffolding and gap repair.

Following MaSuRCA author’s recommendation^8^, we have turned off the *frgcorr* module and provided raw paired-end and mate-pair read libraries for in-built preprocessing with the QuorUM error corrector^13,15^. The initial genome assembly size estimated with the Jellyfish assembler module was 938 Mb. After the MaSuRCA pipeline processing we have polished the assembly with one round of Pilon v1.22, which attempts to resolve assembly errors and fill scaffold gaps using preprocessed reads mapped to the assembly^16^. Leftover contaminants were filtered during the processing of the genome submission to the NCBI database. Statistics of the resulting assembly were generated using bbmap stats toolkit v37.32^17^ and are presented in Table 2.

**Table 2:** DT genome assembly statistics and completeness.

Genome assembly statistics	
Total scaffolds	27,639
Total contigs	36,005
Total scaffold sequence	735.303 Mb
Total contig sequence	725.703 Mb
Gap sequences	1.306%
Scaffold N50	340.819 kb
Contig N50	133.131 kb
Longest scaffold	3.085 Mb
Longest contig	995.155 kb
Fraction of genome in >50 kb scaffolds	88.3%
BUSCO genome completeness score	
Complete	91.5%
Single	87.0%
Duplicated	4.5%
Fragmented	3.6%
Missing	4.9%
Total number of Actinopterygii orthologs	4,584

The resulting 735 Mb assembly had a scaffold N50 of 341 kb, the longest scaffold being more than 3 Mb. To assess the completeness of the assembly we used BUSCO v3^18^ with the Actinopterygii ortholog dataset. In total, 91.5% of the orthologs were found in the assembly.

### Transcriptome sequencing and annotation

**Fig. 3:**
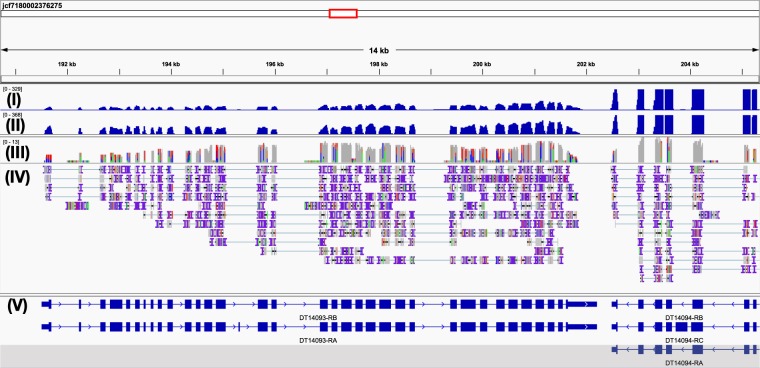
IGV screenshot of the *dnmt1* locus in the DT genome assembly, with short-read RNA coverage, mapped Nanopore cDNA-seq reads and alternative splicing annotation. Tracks from top to bottom: (I) adult RNA-seq coverage, (II) 3 dpf RNA-seq coverage, (III) Nanopore cDNA-seq coverage, (IV) Nanopore cDNA-seq read mapping and (V) annotation with alternative splicing isoforms.

We used three sources of transcriptome evidence for the DT genome annotation: (i) assembled poly-A-tailed short-read and raw Nanopore cDNA sequencing libraries, (ii) protein databases from sequenced and annotated fish species and (iii) trained gene prediction software. For Nanopore cDNA sequencing we extracted total nucleic acids from 1–2 dpf embryos using phenol-chloroform-isoamyl alcohol extraction followed by DNA digestion with DNAse I. The resulting total RNA was converted to double-stranded cDNA using poly-A selection at the reverse transcription step with the Maxima H Minus Double-Stranded cDNA Synthesis Kit (ThermoFisher). The double-stranded cDNA sequencing library was prepared and sequenced in the same way as the genomic DNA with MinKNOW v1.13.1, resulting in 190 Mb sequence data distributed over 209k reads. These reads were filtered to remove 10% of the shortest ones. For short-read RNA-sequencing, we have extracted total RNA with the TRIzol reagent (Invitrogen) from 3 dpf larvae and from adult fish. RNA was poly-A enriched and sequenced as 100 bp paired-end reads on the BGISEQ-500 platform. After preprocessing the library sizes were 65.4 million read pairs for 3 dpf larvae and 64.3 million read pairs for adult fish specimens (Table 1). We first assembled the 100 bp paired-end RNA-seq reads *de novo* using Trinity v2.8.4 assembler^19^. This produced 222448 contigs with an N50 length of 3586 bp, clustered into 146103 “genes”. BUSCO transcriptome analysis revealed 96% of complete Actinopterygii orthologs in the Trinity assembly. These contigs, together with the Nanopore cDNA reads and proteomes of 11 fish species from Ensembl^20^ were used as the transcript evidence in MAKER v2.31.10 annotation pipeline^21^. Repetitive regions were masked using a *de novo* generated DT repeat library (RepeatModeler v1.0.11)^22^. The highest quality annotations with average annotation distance (AED) < 0.25 were used to train SNAP^23^ and Augustus^24^ gene predictors. Gene models were then polished over two additional rounds of re-training and re-annotation. The final set of annotations consisted of 24,097 protein-coding gene models with an average length of 13.4 kb and an average AED of 0.18 (Table 3). We added putative protein functions using MAKER from the UniProt database^25^ and protein domains from the interproscan v5.30–69.0 database^26^. tRNAs were searched for and annotated using tRNAscan-SE v1.4^27^. The BUSCO transcriptome completeness search found 86% of complete *Actinopterygii* orthologs in the annotation set. An example Interactive Genomics Viewer (IGV) v2.4.3^28^ window with the *dnmt1* gene is shown on Fig. 3, demonstrating the annotation and RNA-seq coverage.

**Table 3:** DT transcriptome annotation statistics.

Total protein-coding gene models	24,097
Total functionally annotated gene models	21,491
Gene models with AED <0.5	95%
Mean AED	0.18
BUSCO annotation completeness score	
Complete	86.3%
Single	80.6%
Duplicated	5.7%
Fragmented	7.1%
Missing	6.6%
Total number of Actinopterygii orthologs	4,584

## Data Records

Raw sequencing libraries and genome and transcriptome assemblies are deposited to NCBI SRA as part of the BioProject SRP136594^29^.

The genome assembly with gene and transcript annotations has been deposited at GenBank under the accession number SRMA00000000^30^ (the version described in this paper is SRMA01000000), as well as on figshare in FASTA/GFF3 format^31^. The Trinity transcriptome assembly has been deposited at NCBI TSA under accession number GHNV00000000^32^ (the version described in this paper is GHNV01000000), as well as on figshare^31^.

Kmergenie-generated kmer abundance histograms and a summary report together with the genome size estimation are deposited at figshare^31^.

MAKER pipeline annotation output GFF3 file containing evidence mapping, identified repetitive elements and gene models, MAKER-predicted transcripts and proteins, IGV-compatible short-read and long-read RNA-seq coverage, raw sequencing read library FASTQC quality analysis report and intron orthology data together with their custom analysis code are available on figshare^31^.

## Technical Validation

### DT and zebrafish intron size distributions

The predicted genome size of DT is around one half of the zebrafish reference genome^33^. *Danionella dracula*, a close relative of DT, possesses a unique developmentally truncated morphology^34^ and has a genome of a similar size (ENA Accession Number GCA_900490495.1). In order to validate our genome assembly, we set out to compare the compact genome of DT to the zebrafish reference genome.

**Fig. 4:**
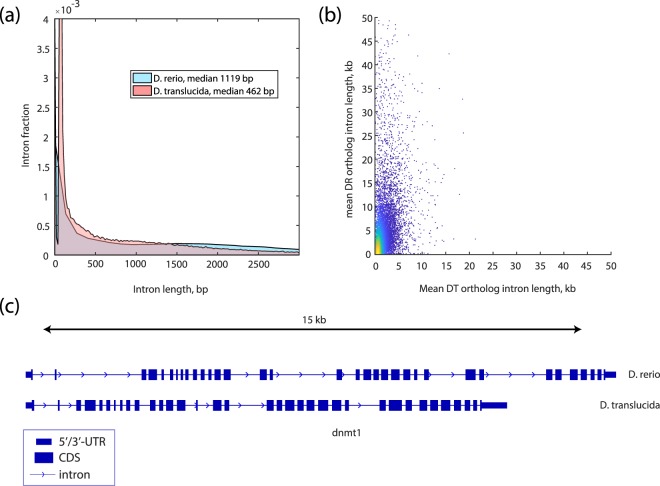
Intron size distribution in DT (red) in comparison to zebrafish (DR, blue). (**a**) Intron size distribution of all transcripts in DR and DT. (**b**) Intron size relationship for identified DR-DT orthologous proteins. (**c**) A comparison of *dnmt1* orthologous loci in both fish. **5**′/**3**′ **UTR**, untranslated regions; **CDS**, coding sequence.

Changes in the intron lengths have been shown to be a significant part of genomic truncations and expansions, such as a severe intron shortening in another miniature fish species, *Paedocypris*^35^, or an intron expansion in zebrafish^36^. We therefore compared the distribution of total intron sizes from the combined Ensembl/Havana zebrafish annotation^20^ to the MAKER-produced DT annotation (Fig. 4a). We found that the DT intron size distribution is similar to other fish species investigated in ref.^35^ which stands in stark contrast to the large tail of long introns in zebrafish. Median intron length values are in the range of the observed genome size difference (462 bp in DT as compared to 1,119 bp in zebrafish).

To investigate the difference in intron sizes on the transcript level, we compared average intron sizes for orthologous protein-coding transcripts in DT and zebrafish. We have identified orthologs in DT and zebrafish protein databases with the help of the conditional reciprocal best BLAST hit algorithm (CRB-BLAST)^37^. In total, we have identified 19,192 unique orthologous protein pairs. For 16,751 of those orthologs with complete protein-coding transcript exon annotation in both fish we calculated their respective average intron lengths (Fig. 4b). The distribution was again skewed towards long zebrafish introns in comparison to DT. As an example, Fig. 4c shows *dnmt1* locus for the zebrafish and DT orthologs.

## ISA-Tab metadata file

Download metadata file

## Data Availability

Software used for read preprocessing, genome and transcriptome assembly and annotation is described in the Methods section together with the versions used. Custom MATLAB code used for orthology analysis is deposited on figshare^31^.
